# Mixed Sequence Reader: A Program for Analyzing DNA Sequences with Heterozygous Base Calling

**DOI:** 10.1100/2012/365104

**Published:** 2012-06-18

**Authors:** Chun-Tien Chang, Chi-Neu Tsai, Chuan Yi Tang, Chun-Houh Chen, Jang-Hau Lian, Chi-Yu Hu, Chia-Lung Tsai, Angel Chao, Chyong-Huey Lai, Tzu-Hao Wang, Yun-Shien Lee

**Affiliations:** ^1^Department of Computer Science, National Tsing Hua University, Hsin-Chu, Taiwan; ^2^Graduate Institutes of Clinical Medical Sciences, Chang Gung University, No. 259 Wen-Hwa, 1st Road, Kwei-Shan, Tao-Yuan 333, Taiwan; ^3^Institute of Statistical Science, Academia Sinica, Taipei, Taiwan; ^4^Department of Biotechnology, Ming Chuan University, Tao-Yuan, Taiwan; ^5^Department of Obstetrics and Gynecology, Lin-Kou Medical Center, Chang Gung Memorial Hospital, Chang Gung University, Fu-Hsing Street, Kwei-Shan, Tao-Yuan 333, Taiwan; ^6^Genomic Medicine Research Core Laboratory, Chang Gung Memorial Hospital, No. 5, Fu-Hsing Street, Kwei-Shan, Tao-Yuan 333, Taiwan

## Abstract

The direct sequencing of PCR products generates heterozygous base-calling fluorescence chromatograms that are useful for identifying single-nucleotide polymorphisms (SNPs), insertion-deletions (indels), short tandem repeats (STRs), and paralogous genes. Indels and STRs can be easily detected using the currently available In*d*elligent or ShiftDetector programs, which do not search reference sequences. However, the detection of other genomic variants remains a challenge due to the lack of appropriate tools for heterozygous base-calling fluorescence chromatogram data analysis. In this study, we developed a free web-based program, Mixed Sequence Reader (MSR), which can directly analyze heterozygous base-calling fluorescence chromatogram data in .abi file format using comparisons with reference sequences. The heterozygous sequences are identified as two distinct sequences and aligned with reference sequences. Our results showed that MSR may be used to (i) physically locate indel and STR sequences and determine STR copy number by searching NCBI reference sequences; (ii) predict combinations of microsatellite patterns using the Federal Bureau of Investigation Combined DNA Index System (CODIS); (iii) determine human papilloma virus (HPV) genotypes by searching current viral databases in cases of double infections; (iv) estimate the copy number of paralogous genes, such as **β**-defensin 4 (*DEFB4*) and its paralog *HSPDP3*.

## 1. Introduction

The detection of genomic variations is important in studying the relationships between causative genes and diseases and the relationships between predisposing genes and complex trait diseases, such as type 2 diabetes, coronary heart disease, and cancers [1–4]. Structural genomic variations also provide important information about both genetic diversity and human evolution [5]. Human genomic variations include single-nucleotide polymorphisms (SNPs), variable number of tandem repeats (VNTRs), short tandem repeats (STRs, or microsatellites), and copy number variations (CNVs) [6]. Among these genomic variants, there are currently 51,810,853 reference SNPs for the human genome, which include 6,516,668 indel sequences and 5,214 microsatellite markers, according to dbSNP Build 135. For instance, a recent genome sequence of a human individual revealed 292,102 heterozygous indel events and 559,473 homozygous indels [7].

**Figure 1:**
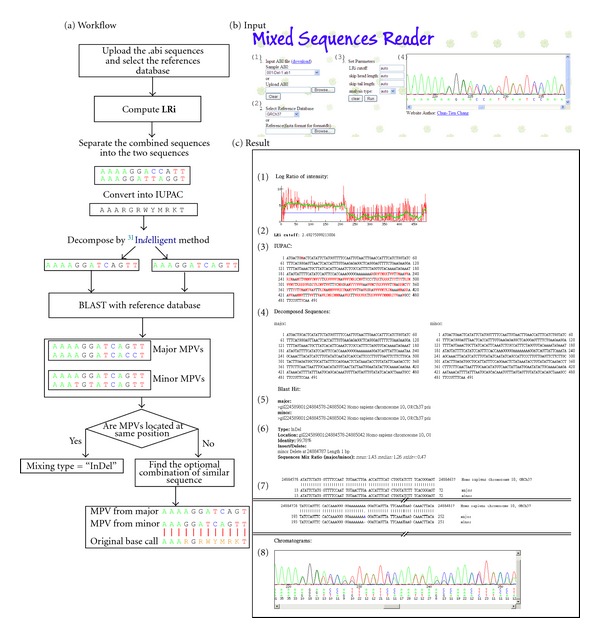
Workflow and user interfaces (input and result output) of the Mixed Sequences Reader (MSR). A flowchart describing the MSR workflow is shown in the left-side panel (a); the input interface of MSR is shown in the right-side panel (b); the result output is shown in panel (c). To input data, users can upload  .abi sequences (b-1) and select the desired reference sequences (b-2). MSR defines an LRi (log ratio of intensity) value for each sequence as the log ratio of the two intensities of the combined signal peaks. On the basis of the smooth LRi curve (c-1), the LRi can be calculated (c-2); this value is used to separate the combined sequences into two sequences and then convert them into IUPAC code (c-3). The IUPAC codes are decomposed by In*d*elligent method into major and minor sequences (c-4). The major and minor sequences are BLASTed against a set of reference sequences to obtain the major and minor most possible variances (MPV) (c-5). If the MPVs are located at same position, the variant type is defined as an “indel”; if they are at different positions, the variant is defined as a “mixed” type (c-6). The variant type is then reported (c-7). The chromatographs of the analyzed sequences are shown in (b-4). The combination of major and minor sequences is shown in (c-8). Users have the option to define the LRi cut-off value, sequence type, and the ignored head and tail sequence lengths (b-3).

**Figure 2:**
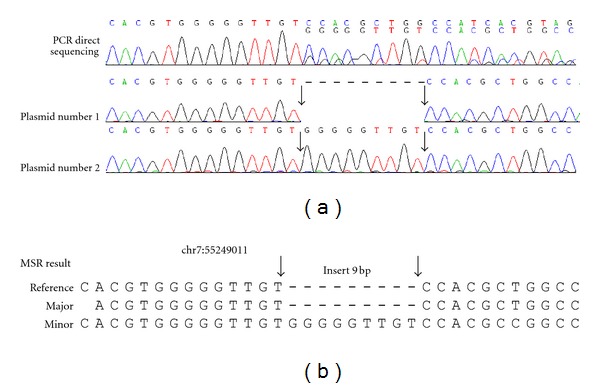
Experimental confirmation of the Indel identified by Mixed Sequence Reader. A 9-bp insertion at chromosome 7:55249011 was detected by Mixed Sequence Reader from a directly sequenced PCR product. (a) PCR direct sequencing chromatography trace, (b) MSR results. The PCR products were cloned, and at least ten single colonies were analyzed by DNA sequencing. One plasmid contained the wild type sequence (plasmid 1); whereas the other plasmids contained an insertion of “GGGGGTTGT,” as shown in plasmid 2 (a). The insertion sequence is shown between two arrows.

**Figure 3:**
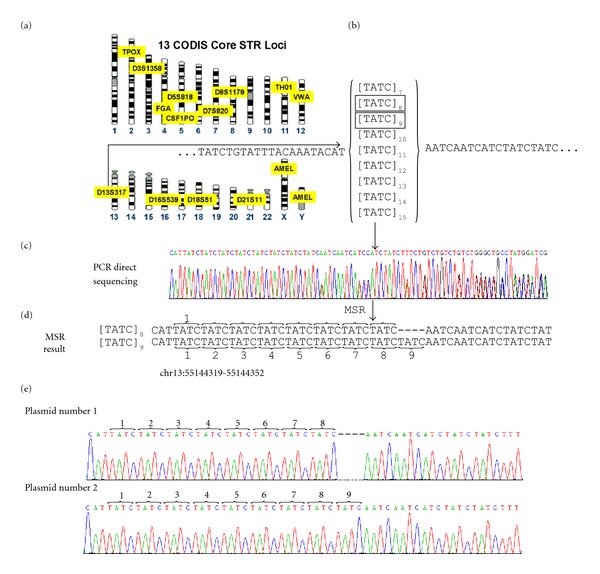
Experimental confirmation of one CODIS-STR locus identified by the Mixed Sequencer Reader. (a) The FBI CODIS Core STR Loci map (adapted from http://www.cstl.nist.gov/strbase/fbicore.htm). The D13S317 locus (chromosome 13:55144219-55144352 region) was randomly selected for analysis in this study. (b) The common repeat structure in the D13S317 locus in each person represents the combination of 2 structures in the CODIS database. (c) The direct sequencing chromatography trace of a PCR product containing the D13S317 locus at chromosome 13:55144219-55144352. (d) The MSR results indicated the presence of 8 and 9 copies of (TATC) within the individual. (e) The sequences of cloned PCR products were consistent with the MSR prediction. One of plasmid contained 8 copies of (TATC) (e.g., plasmid 1); whereas the other plasmids contained 9 copies of [TATC] (e.g., plasmid 2).

**Figure 4:**
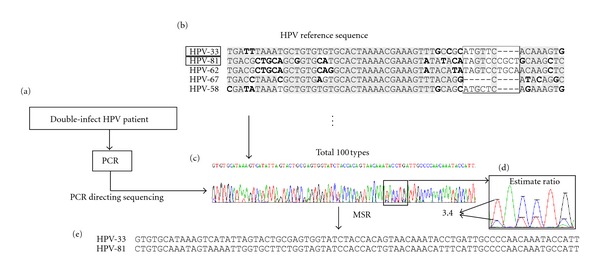
Application of MSR in identifying double-infection of HPV in a cervical cancer sample. (a) A sample infected with two strains of HPV was selected according to HPV array results. The HPV sequences in the specimen were amplified with a pair of PCR primers specific to the L1 region. (b) Alignment of the L1 sequences of several HPV genotypes. (c) PCR direct sequencing results showed a mixed chromatography trace representing two HPV genotypes. (d) MSR estimated the LRi ratio between the major/minor sequences. (e) The MSR output showed that the sample was infected with HPV genotypes 33 and 81.

Genomic variants are frequently identified with heterozygous base-calling fluorescence chromatogram data generated from the direct sequencing of genomic PCR products using the dye-terminator method with Applied Biosystems (ABIs) autosequencers, such as models 3700 or 3730. Several groups have developed programs to analyze heterozygous base-calling fluorescence chromatogram data. For instance, ShiftDetector is a program for detecting shift mutations and calculating a probability score for sequences reconstructed from  .abi sequence files [8]. The In*d*elligent program uses dynamic programming optimization to decode the heterozygous indels with International Union of Pure and Applied Chemistry DNA code system (IUPAC code) [9, 10]. The CHILD (CHromatogram In/Del Location and Detection) program was specifically designed to detect indels in DNA mixtures where one variant is rare, and it can also estimate the ratio of two variants [11]. Generally speaking, all of the currently available programs can be applied for the analysis of indel genomic variants even without reference sequences. However, the alignment of heterozygous base-calling fluorescent data with a reference database can be used to detect the physical position of indel within the genome. Even so, some heterozygous indels may not be easily visualized (Figures 1(b-4) and 2(a)).

Short tandem repeats (STRs) are the most important markers in forensic genetic analysis, and several commercial kits for STR analysis are available [12, 13]. Two-nucleotide repeats are the most prevalent STRs, while repeats with more nucleotides (*n* = 3~6) are less common in the human genome. Among the more than 5000 human STR markers, the Federal Bureau of Investigation (FBI) has included thirteen loci in its Combined DNA Index System (CODIS) database, which contains information from more than 6,384,379 individuals [14–16]. Current STR genotyping uses multiplex PCR with fluorescent STR primers to amplify genomic regions containing VNTRs or STRs, and the PCR products are then separated with capillary electrophoresis, which distinguishes fragments by length but does not display the actual sequence [17]. Nevertheless, sequencing methods remain very useful for analysis of STRs because they reveal the actual sequences of STRs [18], although the number may be too ambiguous to interpret (Figure 3(c)). To resolve this ambiguity, DNA cloning is often required to identify the different sequences of the two alleles. Currently, no program is able to analyze microsatellite repeat units (or CODIS) directly using the chromatogram trace data, even though the chromatogram trace profiles of heterozygous microsatellites are similar to those of heterozygous indels with 2 or more nucleotide deletions (Supplementary Figure  1 in Supplementary Materials available online at doi:10.1100/2012/365104).

Some genotypes of HPV are oncogenic. Therefore, routine screening and genotyping of HPV in women are crucial for prevention of cervical cancer. Current HPV detection methods are either using PCR techniques or HPV genotyping array system [19]. About 90% of cervical cancer tissues are infected by a single HPV genotype, while about 10% of specimens by two or more types of HPV. In the single HPV-infected samples, the viral genotype can be easily identified by genotyping array or PCR. For those with mixed infection by double types of HPV, mixed chromatogram traces are observed, in a similar way to those of SNP and indel sequences (Figure 4(b)).

**Figure 5:**
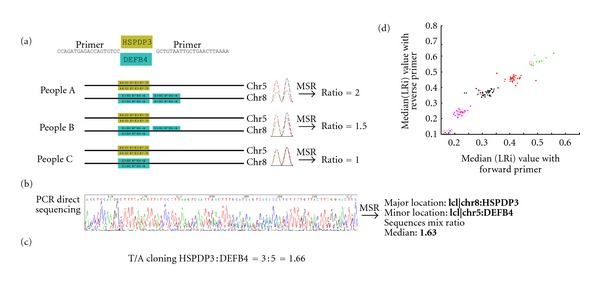
Experimental confirmation of *HSPDP3* and *DEFB4* copy numbers identified by Mixed Sequence Reader. (a) The primers used to amplify the *DEF4B* and *HSPDP3* genes in the human genome. The LRi ratio between *DEFB4* and *HSPDP3* was calculated for each chromatography trace. The copy number of *HSPDP3* is constant in the human genome (*n* = 2). (b) The heterozygous chromatography trace of PCR products comprising both *HSPDP3* and *DEFB4*. The median *DEFB4*/*HSPDP3* ratio was 1.63, which was estimated by MSR program. (c) The PCR product from (b) was cloned, and at least twenty colonies were picked for DNA sequencing to calculate the ratio between *DEFB4* and *HSPDP3*. The observed ratio was 1.66, compatible with that derived from MSR. (d) Comparison of median LRi values from the forward and reverse primers. Using the k-means algorithm, pairs of median LRi values were clustered into 5 groups corresponding to different copy numbers (2, 3, 4, 5, and 6). Five samples (marked with circles) were cloned into the T and A vectors to validate the *DEFB4 *copy number.

CNVs, another type of genomic variation, are segments of DNA with variable copy numbers; the length of a single CNV may range from one kilobase to several megabases [20]. One well-known CNV occurs in the *β*-defensin 4 locus (*DEFB4*), which is known to have copy numbers that range from two to seven. The CNV of the defensin genes is associated with increased susceptibility to infectious diseases, autoimmune diseases, inflammatory disorders, Crohn's disease, and certain cancers [21, 22]. Array-based comparative genomic hybridization (aCGH) is used to detect genomic CNVs. Other methods for CNV validation include multiplex amplifiable probe hybridization combined with restriction enzyme digest variant ratios (MAPH/REDVRs) [23, 24], multiplex ligation-dependent probe amplification (MLPA) [25, 26], paralog ratio test (PRT) [24, 27, 28], and real-time polymerase chain reaction [29]. The PRT method uses a pair of primers to amplify two paralogous sequences, which are then separated with capillary electrophoresis. By detecting the ratio between the chromatographic intensities of the two PCR products, PRT can estimate the copy number of the CNV [24, 27, 28]. However, the chromatogram traces of the PCR products derived from paralogous genes are often too heterozygous to be analyzed with any currently available programs (Figure 5(b)).

To address the above issues, we have developed a program, Mixed Sequencer Reader (MSR), which can be used to identify indels, microsatellite copy numbers, and CODIS combinations. On the basis of the In*d*elligent method, the heterozygous sequences are identified as two distinct sequences, which are further aligned with reference genomic sequences to provide information about the physical position of indels, copy number or types of STR, and paralogous genes. We also applied this program to identify double HPV infections in cervical cancer tissues and estimate the copy number of paralogous human genes (e.g., the *β*-defensin 4 gene *DEFB4 *and its paralog *HSPDP3*). The software is freely available at http://MSR.cs.nthu.edu.tw/.

## 2. Materials and Methods

### 2.1. Mixed Sequence Reader (MSR)

The MSR program was developed to detect heterogeneity in chromatographic traces, determine the physical positions of the detected variants in the human genome, and identify the type of genomic variation present. The algorithm used in MSR was modified from that of In*d*elligent, but MSR is designed to use reference database alignment. The analytic steps used in MSR are described below and shown in Figure 1(a).

#### 2.1.1. Importing the DNA Sequence Chromatographs and Selecting the Reference Database

The imported files are chromatography traces in the  .abi format. The base peak positions, quality values, and four channel signals (A, C, G, T) recorded in the  .abi file are extracted and analyzed to identify the major and the minor signals at each base location (Figures 1(b-1) and 1(b-4)). Users can select the desired reference database (Figure 1(b-2)).

#### 2.1.2. Defining the Log Ratio of Intensity (LRi)

The LRi value for each base position is defined as the log ratio of the chromatographic intensities of two combined sequences. The formula for LRi is:

(1) $$\text{LRi}=\log_{2}\left(\frac{\text{major fluorescence intensity}}{\text{minor fluorescence intensity}}\right).$$

If the sequence position contained only one major band (no heterogeneity in the chromatographic trace), the value of LRi should be infinite (log⁡_2_(1/0)). If the DNA sequences contain two heterogeneous chromatographic traces with equal intensities, the LRi should be 0 (log⁡_2_(1/1)). After the  .abi file is imported, users can define an LRi cut-off value (Figure 1(b-3)), or the MSR program can automatically set the cut-off value using smooth LRi (Figure 1(c-1), green line). For example, in the results shown in Figure 1(c-1), the shift of signal intensity at 212 bp was detected by the MSR program and considered an “indel”-type heterozygosity. The LRi values of “indel” sequences are higher than the cutoff value for sequences without heterogeneous fluorescence chromatography traces, so the LRi line drops when heterogeneous fluorescence traces are identified. When there is no obvious shift of the LRi line, the sequences might be a “mixed” type, and the LRi cutoff value is automatically set at 2.0 by the MSR program. The sequences of either “indel” or “mixed” heterozygosities are then displayed in IUPAC code (Figure 1(c-3)).

#### 2.1.3. Decomposing the IUPAC Code Using the Indelligent Algorithm

In the In*d*elligent algorithm, dynamic programming is used to convert the IUPAC code into two nucleotides (i.e., M is converted into A/C, W into A/T, Y into C/T, K into G/T, and S into G/C) [9, 10]. For ambiguous bases that cannot be decomposed with In*d*elligent, major and minor sequences are assigned according to the intensities of the corresponding fluorescence signals (Figure 1(c-4)).

#### 2.1.4. Finding the DNA Sequence of the Most Possible Variances (MPVs) by BLAST

The major and minor DNA sequences derived from Section 2.1.3 were further analyzed by BLASTN against the databases that were built into the MSR program (GRCh37 primary reference assembly or CODIS, HPV, *DEFB4*/*HSPDP3* reference sequences) or user-defined reference sequences (Figure 1(b-2)) to detect the most possible variances (Figure 1(c-5)). The major and minor sequences were BLASTed against reference sequences to obtain the major and minor MPVs. Once the physical positions of the major and minor MPVs were identified, the sequences were categorized as indels (Figure 1(c-6)) or considered as a “mixed” type. For “mixed” type heterogeneities, MSR continues with the following procedures.

#### 2.1.5. Deriving the Optimal Combinations

Each top M (major) MPV and top N (minor) MPV are combined pairwise into IUPAC code, resulting in (M × N) combinations. Each IUPAC code combination is aligned to the original signal IUPAC code (Figure 1(c-3)), and the (M × N) IUPAC code combinations with the highest scores are identified as the optimal MPV combinations by the MSR program (Figure 1(c-7)). Using the selected reference database, such as the default MSR human genome sequence database or a user-imported database, the MSR program identifies the genotypes of the mixed sequences.

#### 2.1.6. Calculating the Ratio between the Major and Minor Sequences

For all heterogeneous bases, the LRi values were calculated as described in Section 2.1.2. MSR calculated the medium LRi value of heterogeneous nucleotides as the “Sequence Mix Ratio” (example shown in Figure 4(d)). The Sequence Mix Ratio is proportional to the signal ratio between the major and minor sequences.

### 2.2. DNA Cloning

To validate DNA sequences, PCR products amplified from the experimental samples were selected for cloning experiments to confirm the variations. The PCR products were cloned into the pCRII-TOPO cloning system (Invitrogen) according to the manufacturer's recommendations. The cloned DNA sequences were then analyzed with an ABI 3770 autosequencer.

### 2.3. Determination of the *β*-Defensin 4 (*DEFB4*) Copy Number

All DNA samples used in this study were unlinked from clinical information, and the DNA collection was approved by the Institutional Review Board (IRB) of Chang Gung Memorial Hospital (CGMH) (#99-0229B, IRB#100-2900A3). Genomic DNA samples from 100 normal individuals were tested for CNVs of the paralogous *DEFB4*/*HSPDP3 *genes. *DEFB4 *and its paralog, the *HSPDP3* pseudogene, were amplified from 50 genomic DNA specimens. PCR using a previously reported pair of primers [27] amplifies two products of similar size, one from the *DEFB4* gene on chromosome 8 and the other from the* HSPDP3* pseudogene on chromosome 5 (the copy number of *HSPDP3* is always 2). These PCR products were directly sequenced (see Figure 5(b) for an example). For each peak in the chromatogram trace, we calculated the LRi value, which indicated the *DEFB4*/*HSPDP3* ratio. For each sample, the median values of all heterogeneous sequences were used to determine the copy number of the *DEFB4* gene. The k-means method was used to partition the different copy numbers.

## 3. Results

### 3.1. The Mixed Sequences Reader Interface

The data import interface of the Mixed Sequence Reader is divided into three parts (Figure 1(b)): (1) data import, (2) reference database selection, and (3) MSR parameter settings. At the data import step, users can import an  .abi file into MSR (Figure 1(b-1)). Users can also test the performance of MSR using 260 sample  .abi files (Figure 1(b-1)), some of which were experimentally validated in this study. After the  .abi files are imported, users can preview the chromatography data by moving the cursor in the right side panel (Figure 1(b-4)). Users can then either select the reference database to use (GRCh37, CODIS, HPV, *DEFB4*/*HSPDP3*) or import their own reference sequences in FASTA format (Figure 1(b-2)). Then, users can define the LRi cutoff value, sequence type and specify the ignored head and tail sequence lengths (Figure 1(b-3)). Then, users click the “Run” button to execute the MSR program. The results of a sample analysis are shown in Figure 1(c).

### 3.2. Indel and Short Tandem Repeat Sequences

We first analyzed indel sequences with MSR (Figure 2(b)). From the NCBI dbSNP database, we selected 6 indel sites of high heterozygosity, PCR amplified the corresponding genomic DNA, and directly sequenced the PCR products to obtain  .abi files. Fourteen  .abi files were successfully analyzed by MSR (Supplementary Table  1). To validate the MSR results, the predicted indel sequences were confirmed by cloning the PCR products, followed by sequencing of the plasmid DNAs. For example, one heterozygous chromatography trace was predicted to contain a nine nucleotide insertion at human chr7:55249011 (Figure 2(b)). Although similar results were also obtained when the sequences were analyzed with In*d*elligent or other programs, the MSR was able to identify the physical position of the indel by BLASTing the sequences against the GRCh37 database (Figure 2(b)). The experimental results confirmed the results predicted by MSR (Figure 2(a)).

 We also selected several simple short tandem repeats (STRs) from the NCBI dbSNP database. Three STR-containing regions of the genome were amplified by PCR, sequenced, and analyzed with MSR (13  .abi files of STR in Supplementary Table  2). Among the 13  .abi files analyzed, the PCR products corresponding to 2  .abi files were further cloned and validated by single-plasmid DNA sequencing. The number of each repeats were also defined by MSR (Supplementary Figure  1).

### 3.3. The Repeat Structure of a Short Tandem Repeat in the CODIS Database

 Because CODIS is the largest STR database currently available, the repeat patterns of the 13 loci in CODIS were documented (Figure 3(a)) [14, 15]. The D13S317 locus ([TATC]_7–15_) was amplified by PCR using specific primers (Supplementary Table  3), and the resulting PCR products were directly sequenced (Figures 3(b) and 3(c)). According to the MSR results, the D13S317 locus genotype in the tested individual should be [TATC]_8_/[TATC]_9_ (Figure 3(d)). The PCR products were further cloned and sequenced, and the results confirmed the MSR prediction (Figure 3(e)). Similar results were obtained when the same  .abi file was analyzed using the In*d*elligent program.

 Some STRs in the CODIS database contain complex repeat structures, such as the D21S11 locus with the 65-repeat structure [TCTA]_4–11_[TCTG]_3–14_[TCTA]_0–3_[TA]_0-1_ [TCTA]_3_TCA[TCTA]_2 _TCCATA [TCTA]_6–15_. The alignment of the two sequences in the sample 211-CODIS-D21S11-3  .ab1 file detected two 4-bp gap structures (Supplementary Figure  2). Because of the complexity of the CODIS STR repeat structure, its pattern was not easily solved using the In*d*elligent program without a reference database. However, the optimal STR repeat structure was successfully identified by the MSR program using the reference databases. A total of 29 sequences for 10 CODIS STR sites were analyzed with MSR, and the results are shown in Supplementary Table  4.

### 3.4. Infection with Two Genotypes of HPV in Cervical Cancer Specimens

 Another type of “mixed” sequence is represented by viral coinfection in the same specimen. For example, double HPV genotypes within one sample could be analyzed by EasyChip using probes designed to amplify the variable regions that are unique to each HPV genotype [19]. To validate the microarray data, dually infected specimens were amplified at the L1 region of HPV and the resulting PCR products were sequenced directly (Figure 4(c)). The alignment of sequences representing the different genotypes of HPV revealed both indels and SNPs (Figures 4(b) and 4(c)). One of the samples was identified by MSR as coinfected with HPV-33 and HPV-81, compatible with the EasyChip results (Figure 4(e)). In addition, the ratio between the major (HPV-33) and minor (HPV-81) sequences calculated by MSR could be used to estimate the relative ratio of the two genotypes of HPV. The ratio between HPV-33 and HPV-81 was 3.4 : 1. Genotype-specific PCR confirmed the results of MSR prediction for all 7 specimens that were defined by EasyChip as infected with multiple HPV strains (Supplementary Table  5).

### 3.5. Copy Number Variations of Paralogous Genes

We also applied MSR to detect the copy number of the *DEFB4* gene, which is well known for its multiple copy number variations. The PCR primers used amplified *DEFB4* and its paralog, the pseudogene *HSPDP3 *(Figure 5(a)) [27]; thus, the chromatography traces generated by PCR direct sequencing were very heterozygous (Figure 5(b)) because the chromatographs contained sequences from both chromosome 5 (*HSPDP3* as reference, copy number *n* = 2) and chromosome 8 (*DEFB4*, often variable copy numbers). The LRi value of each chromatography peak was used to estimate the ratio between* DEFB4*/*HSPDP3* and calculate the *DEFB4* copy number (Figure 5(a), right-side panel). The med(LRi) was defined as the median LRi value in all heterogeneous sequences. The scatter plot of the med(LRi) values of the DNA sequences amplified by the forward and reverse primers for 98 individual DNA samples is shown in Figure 5(d). The five groups shown in different colors were partitioned with the K-means clustering algorithm. To confirm these results, 5 specimens were randomly selected and the PCR products were cloned into the pCRII-TOPO cloning system for single-plasmid sequencing (Figure 5(d), circles; Supplementary Table  6). For each specimen, at least 20 clones were sequenced to calculate the* HSPDP3* and *DEFB4* ratio. All of the cloning-sequencing results were identical to the results predicted by MSR.

## 4. Discussion

Structural variations in the human genome are clinically important [6]. For instance, CNVs in *DEFB4* are associated with susceptibility to infectious disease, autoimmune, inflammatory disorders, and even cancers [5, 20]. The copy numbers of the genes *CCL3 L1*, *CCL4 L1*, and *TBC1D3* vary between 0 and 10 copies, and such variations have been found to be associated with susceptibility to HIV-1 [30, 31]. In addition, microsatellite markers are used as indicators for global genome stability and are especially useful in genomic research of cancer [32]. In short, structural variations shape the genome and determine disease susceptibility at the individual level. Therefore, analytical methods that can detect structural genomic variations are acutely needed to study the relationship between genomic variations and disease.

The MSR program introduced in this study can be used to directly analyze heterozygous base-calling chromatographs to detect multiple structural variations, including SNPs, microsatellites, and CNVs, in the human genome. The fluorescence intensity of chromatographs has already been used to detect SNPs [33], but we have extended this analysis of heterozygous base-calling chromatography to explore more structural variations in the human genome. The accuracy of the MSR predictions was validated by other methods. Our analyses show that MSR can also be used to identify double infections by different genotypes of human papilloma virus (HPV) in cervical cancer tissues [34, 35]. It is worth testing whether MSR can be used to determine the presence of multiple viral infections in other cancer tissues [34, 35]. The ability to identify dual viral infections is limited because the reference sequences for these viruses often mutate rapidly, especially for RNA viruses [36]. Therefore, to improve this program for the aforementioned applications, we would need more clinical specimens to challenge this software.

The MSR program does have some limitations, however. First, the majority of structural variations in the human genome, which can be readily identified with MSR, are small indels. On the other hand, deletions of large fragments are likely better analyzed by array-based comparative genomic hybridization methods (aCGH) [37]. Second, some of the heterozygosity identified by base-calling chromatography may be caused by the formation of DNA secondary structures (likely in the GC-rich or AT-rich regions) that result in band compression; these sequences are difficult to analyze with MSR. Third, the STRs in the human genome are not yet fully characterized. Therefore, the MSR may not be able to predict all STRs. In this study, we used some types of STRs as examples of the potential of MSR, but more samples are needed to analyze all 13 core loci in the CODIS database and other STRs. Fourth, MSR and all other web sources only provide tools to analyze or predict structural variations in the human genome. We should rely on experimental data to confirm these results. Fifth, the MSR program is designed to read only fluorescence chromatography tracings derived from the ABI 3730 autosequencer, but not for those generated by the recently developed ultrahigh-throughput sequencers, such as Roche 454, ABI SoLid, and Illumina/Solexa.

A comparison of the features of the MSR program and other currently available DNA sequence analysis software is summarized in Table 1. All of the available programs can detect indels. Most of the programs can read files in  .abi format, with the exception of In*d*elligent, which only processes IUPAC code data. Only MSR can map sequence data to a reference database and report the most possible mixed sequence. MSR and CHILD can estimate the ratio of sequences in a mixture. Only PolyScan can process next-generation sequencing data [38].

 In conclusion, we have developed a user-friendly web-based program, Mixed Sequences Reader (MSR), to analyze heterozygous fluorescent chromatographs derived from an autosequencer. Using this program, several types of human genomic variations, including SNPs, indels, and CNVs of microsatellites or genes can be detected from a single DNA sequence read. Furthermore, MSR is useful for detecting viral infection with double genotypes in clinical specimens.

## Supplementary Material

Supplementary Table 1: The filename, LRi cutoff value, and physical position in samples used for InDel analysis in this study.
Supplementary Table 2: The filename, LRi cutoff value and repeat structure in samples used for microsatellite analysis in this study.
Supplementary Table 3: The primer paris designed for amplification of 10 CODIS locus in this study.
Supplementary Table 4: The filename, LRi cutoff value and repeat structure in samples analyzing CODIS pattern in this study.
Supplementary Table 5: The filename, LRi cutoff value, and HPV genotype in dual infection cervical cancers samples in this study.
Supplementary Table 6: The copy number ratio of DEFB4/HSPDP3 predicted by MSR program and T/A cloning experiment.
Supplementary Figure 1: (A) Chromatography trace of one Indel [CA] STR repeat structure and those of plasmid cloning, (B) MSR analyzed results are shown as the IUPAC code and decomposed sequence.
Supplementary Figure 2: (A) Chromatography trace of a combined two CODIS D21S11 Locus subtype, (B) there were two 4-bp gaps in the alignment of two subtypes in CODIS D21S11 Locus.

## Figures and Tables

**Table 1:** Feature comparison between MSR and other currently available DNA sequencing software.

Feature/software	MSR	In*d*elligent	ShiftDetector	CHILD	PolyScan
Directly reads trace files (abi format)	Yes	No	Yes	Yes	No
Maps sequence to reference database	Yes	No	No	No	No
Detects indels	Yes	Yes	Yes	Yes	Yes
Decomposes 2 mixed sequences from a single trace	Yes	Yes	No	Yes	No
Estimates the ratio of 2 mixed sequences in one trace	Yes	No	No	Yes	No
Accepts NGS data	No	No	No	No	Yes
