# Supplementary material for: Mixed Sequence Reader: A Program for Analyzing DNA Sequences with Heterozygous Base Calling
Source: ScientificWorldJournal. 2012 Jun 18;2012:365104. doi: 10.1100/2012/365104 (PMC3385616; doi:10.1100/2012/365104)
Supplement: Supplementary file 1 — Supplementary Table 1: The filename, LRi cutoff value, and physical position in samples used for InDel analysis in this study. Supplementary Table 2: The filename, LRi cutoff value and repeat structure in samples used for microsatellite analysis in this study. Supplementary Table 3: The primer paris designed for amplification of 10 CODIS locus in this study. Supplementary Table 4: The filename, LRi cutoff value and repeat structure in samples analyzing CODIS pattern in this study. Supplementary Table 5: The filename, LRi cutoff value, and HPV genotype in dual infection cervical cancers samples in this study. Supplementary Table 6: The copy number ratio of DEFB4/HSPDP3 predicted by MSR program and T/A cloning experiment. Supplementary Figure 1: (A) Chromatography trace of one Indel [CA] STR repeat structure and those of plasmid cloning, (B) MSR analyzed results are shown as the IUPAC code and decomposed sequence. Supplementary Figure 2: (A) Chromatography trace of a combined two CODIS D21S11 Locus subtype, (B) there were two 4-bp gaps in the alignment of two subtypes in CODIS D21S11 Locus. [file 365104.f1.pdf]

Supplementary Table 1 : The filename, LRi cutoff value, and physical position in samples used for InDel analysis in this study

<table>
<tr><th>Filename</th><th>LRi cutoff</th><th>InDel Type</th><th>Location</th><th>Variation Position</th></tr>
<tr><td>001-Del-1</td><td>2.69</td><td>1bp Delete</td><td>gi|224589801|chr10</td><td>24884787</td></tr>
<tr><td>002-Del-1</td><td>2.65</td><td>1bp Delete</td><td>gi|224589801|chr10</td><td>24884787</td></tr>
<tr><td>003-Del-1</td><td>1.98</td><td>1bp Delete</td><td>gi|224589803|chr12</td><td>51442577</td></tr>
<tr><td>004-Del-1</td><td>2.72</td><td>1bp Delete</td><td>gi|224589803:|chr12</td><td>48237736</td></tr>
<tr><td>005-Del-1</td><td>1.94</td><td>1bp Delete</td><td>gi|224589803:|chr12</td><td>48237736</td></tr>
<tr><td>006-Del-1</td><td>2.50</td><td>1bp Delete</td><td>gi|224589801|chr10</td><td>24884787</td></tr>
<tr><td>007-Del-1</td><td>1.93</td><td>1bp Delete</td><td>gi|224589803:|chr12</td><td>51442577</td></tr>
<tr><td>008-Del-1</td><td>3.00</td><td>1bp Delete</td><td>gi|224589822|chrX</td><td>69176881</td></tr>
<tr><td>009-Del-1</td><td>3.20</td><td>1bp Delete</td><td>gi|224589822|chrX</td><td>69176881</td></tr>
<tr><td>010-Del-1</td><td>3.86</td><td>1bp Delete</td><td>gi|224589801|chr10</td><td>24884787</td></tr>
<tr><td>011-Del-1</td><td>3.85</td><td>1bp Delete</td><td>gi|224589801|chr10</td><td>24884787</td></tr>
<tr><td>013-Ins-9</td><td>3.76</td><td>9bp Insert</td><td>gi|224589819|chr7</td><td>55249011</td></tr>
<tr><td>016-Ins-6</td><td>3.43</td><td>6bp Insert</td><td>gi|224589819|chr7</td><td>55249013</td></tr>
<tr><td>017-Ins-6</td><td>3.58</td><td>6bp Insert</td><td>gi|224589819|chr7</td><td>55249013</td></tr>
</table>

Supplementary Table 2 : The filename, LRi cutoff value and repeat structure in samples used for microsatellite analysis in this study

Filename	Lri cutoff	Repeat Structure	
126-uSat-CA	3.02	**$[CA]_{16}$**	**$[CA]_{15}$**
127-uSat-AC	2.00	**$[AC]_{24}$**	**$[CA]_{22}$**
151-uSat-TG	2.00	$[TG]_{23}$	$[TG]_{22}$
158-uSat-TG	2.00	$[TG]_{18}$	$[TG]_{17}$
159-uSat-TG	2.00	$[TG]_{18}$	$[TG]_{17}$
161-uSat-CA	3.28	$[CA]_{18}$	$[CA]_{17}$
162-uSat-CA	3.13	$[CA]_{18}$	$[CA]_{17}$
163-uSat-TG	2.00	$[TG]_{18}$	$[TG]_{17}$
183-uSat-TG	2.00	$[TG]_{18}$	$[TG]_{17}$
187-uSat-TG	2.00	$[TG]_{18}$	$[TG]_{17}$
188-uSat-CA	2.00	$[CA]_{18}$	$[CA]_{17}$
192-uSat-CA	2.00	$[CA]_{18}$	$[CA]_{17}$
194-uSat-CA	2.00	$[CA]_{23}$	$[CA]_{22}$

Supplementary Table 3 : The primer paris designed for amplification of 10 CODIS locus in this study

Name	primer_F	primer_R	Product Size
msTH01	CTAGTCAGCACCCCAACCAG	TCCCTGAGAAGGTACCTGGA	434 bp
msCSF1PO	GGAACATATGCAAGGCTCAAA	CAGACTCTTCCACACACCACTG	600 bp
msTPOX	CACTGCTCTGTCGTGACTCC	GAGGAAGGGCTGTGTTTCAG	387 bp
msD13S317	TTTGGGTAGGAAAAAGAGTGGA	TTGATGTATTGCAAGCACTTAGTT	482 bp
msD16S539	CAAGCGAAAGTGATGCCATA	ATCTGCCCTCATCAGTGGAC	573 bp
msD5S818	TTTTGAAAATACATGGGAGAAAA	CCATCTGGATAGTGGACCTCA	454 bp
msD3S1358	CAGCGAGACCCCATCTCTTA	AGCTGCCAGGGCACTGCT	391 bp
msD8S1179	CACAGCTATTATCAAAAACCCAGA	GCTGAGTCTGAAGTAAGTAAAACATTG	500 bp
msFGA	TGAAATGCAATCAAACCAACT	GCCTTCCTTTTCCCTCTACTC	600 bp
msD21S11	TGTCTGGCACCCAGTAAAAA	CTTTGGCCTCAAACTGAAGG	486 bp

Supplementary Table 4 : The filename, LRi cutoff value and repeat structure in samples analyzing CODIS pattern in this study

Filename	Lri cutoff	Repeat Structure	Note
200-CODIS-CSF1PO-1	1.64	$[AGAT]_{10}$<br>$[AGAT]_{13}$	
201-CODIS-CSF1PO-2	2.00	$[AGAT]_{12}$<br>$[AGAT]_{12}$	
202-CODIS-CSF1PO-3	2.00	$[AGAT]_{12}$<br>$[AGAT]_{12}$	
203-CODIS-D13S317-1	1.58	$[TATC]_{9}$<br>$[TATC]_{11}$	
204-CODIS-D13S317-2	3.27	$[TATC]_{8}$<br>$[TATC]_{11}$	
205-CODIS-D13S317-3	2.20	$[TATC]_{8}$<br>$[TATC]_{9}$	
206-CODIS-D16S539-1	2.00	$[GATA]_{11}$<br>$[GATA]_{11}$	
207-CODIS-D16S539-2	3.79	$[GATA]_{9}$<br>$[GATA]_{12}$	
208-CODIS-D16S539-3	3.13	$[GATA]_{11}$<br>$[GATA]_{12}$	
209-CODIS-D21S11-1	2.50	$[TCTA]_{6}[TCTG]_{6}[TCTA]_{3}TA[TCTA]_{3}TCA[TCTA]_{2}TCCA\ TA\ [TCTA]_{10}$<br>$[TCTA]_{6}[TCTG]_{6}[TCTA]_{3}TA[TCTA]_{3}TCA[TCTA]_{2}TCCA\ TA[TCTA]_{12}\ TA\ TCTA$	
210-CODIS-D21S11-2	2.67	$[TCTA]_{5}[TCTG]_{6}[TCTA]_{3}TA[TCTA]_{3}TCA[TCTA]_{2}TCCA\ TA\ [TCTA]_{13}$<br>$[TCTA]_{4}[TCTG]_{6}[TCTA]_{3}TA[TCTA]_{3}TCA[TCTA]_{2}TCCA\ TA\ [TCTA]_{12}$	
211-CODIS-D21S11-3	3.45	$[TCTA]_{6}[TCTG]_{5}[TCTA]_{3}TA[TCTA]_{3}TCA[TCTA]_{2}TCCA\ TA\ [TCTA]_{10}$<br>$[TCTA]_{5}[TCTG]_{6}[TCTA]_{3}TA[TCTA]_{3}TCA[TCTA]_{2}TCCA\ TA\ [TCTA]_{11}$	
212-CODIS-D3S1358-1	2.00	$TCTA[TCTG]_{2}[TCTA]_{12}$<br>$TCTA[TCTG]_{2}[TCTA]_{12}$	

213-CODIS-D3S1358-2	2.00	TCTA$[TCTG]_2[TCTA]_{11}$<br>TCTA$[TCTG]_2[TCTA]_{12}$	skip tail 100bp
214-CODIS-D3S1358-3	1.74	TCTA$[TCTG]_2[TCTA]_{13}$<br>TCTA$[TCTG]_3[TCTA]_{13}$	skip tail 100bp
215-CODIS-D5S818-1	2.25	$[AGAT]_{10}$<br>$[AGAT]_{11}$	
216-CODIS-D5S818-2	2.43	$[AGAT]_{10}$<br>$[AGAT]_{14}$	
217-CODIS-D5S818-3	2.37	$[AGAT]_{11}$<br>$[AGAT]_{12}$	
218-CODIS-D8S1179-1	1.97	$[TCTA]_{11}$<br>$[TCTA]_1[TCTG]_1[TCTA]_{12}$	
220-CODIS-D8S1179-3	2.35	$[TCTA]_{11}$<br>$[TCTA]_1[TCTG]_1[TCTA]_{13}$	
221-CODIS-FGA-1	4.44	$[TTTC]_3$TTTT TTCT$[CTTT]_{15}$CTCC$[TTCC]_2$<br>$[TTTC]_3$TTTT TTCT$[CTTT]_{15}$CTCC$[TTCC]_2$	
222-CODIS-FGA-2	2.00	$[TTTC]_3$TTTT TTCT$[CTTT]_{13}$CTCC$[TTCC]_2$<br>$[TTTC]_3$TTTT TTCT$[CTTT]_{13}$CTCC$[TTCC]_2$	
223-CODIS-FGA-3	2.00	$[TTTC]_3$TTTT TTCT$[CTTT]_{14}$CTCC$[TTCC]_2$<br>$[TTTC]_3$TTTT TTCT$[CTTT]_{17}$CTCC$[TTCC]_2$	
224-CODIS-TH01-1	1.71	$[AATG]_7$<br>$[AATG]_{10}$	
225-CODIS-TH01-2	1.36	$[AATG]_7$<br>$[AATG]_9$	
226-CODIS-TH01-3	2.01	$[AATG]_6$<br>$[AATG]_9$	
227-CODIS-TPOX-1	2.02	$[AATG]_8$<br>$[AATG]_{11}$	skip head 100bp

228-CODIS-TPOX-2	2.00	$[AATG]_8$<br>$[AATG]_8$	
229-CODIS-TPOX-3	3.94	$[AATG]_8$<br>$[AATG]_8$	

Supplementary Table 5 : The filename, LRi cutoff value, and HPV genotype in dual infection cervical cancers samples in this study

Filename	LRi cutoff	HPV type
300-HPV-33-81	4.71	HPV 33/81
301-HPV-42-71	2.00	HPV 42/71
302-HPV-52-18	4.50	HPV 52/18
303-HPV-54-67	3.66	HPV 54/67
304-HPV-6-53	5.45	HPV 6/53
305-HPV-53-52	3.56	HPV 53/52
306-HPV-52-70	2.00	HPV 52/70

Supplementary Table 6 : The copy number ratio of DEFB4/HSPDP3 predicted by MSR program and T/A cloning experiment

	T/A Cloning		MSR Result		T/A Cloning	MSR Result
Sample	HSPDP3	DEFB4	HSPDP3	DEFB4	Log2(DEFB4/HSPDP3*2)	Log2(DEFB4/HSPDP3*2)
629	3	5	2	3	2	2
654	3	11	2	6	3	3
658	5	5	2	3	1	1
718	3	14	2	8	3	3
728	3	3	2	3	1	1

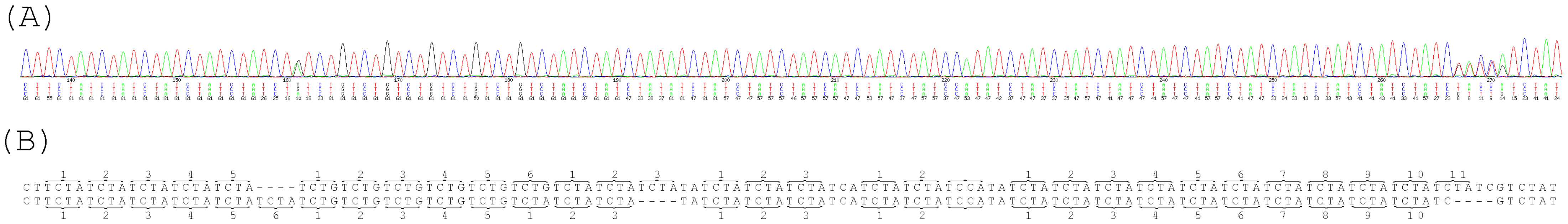

Supplementary Fig. 1 (A) Chromatography trace of one Indel [CA] STR repeat structure and those of plasmid cloning, (B) MSR analyzed results are shown as the IUPAC code and decomposed sequences

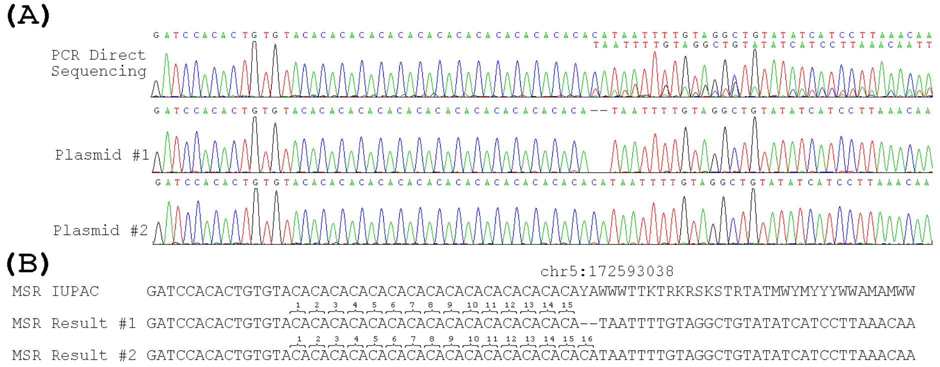

Supplementary Fig. 2 (A) Chromatography trace of a combined two CODIS D21S11 Locus subtype, (B) there were two 4-bp gaps in the alignment of two subtypes in CODIS D21S11 Locus.
